# Influence of Connective Architectures of Inlaid Weft-Knitted Spacer Fabric on Compression, Impact Force Absorption, and Vibration Isolation

**DOI:** 10.3390/polym18020151

**Published:** 2026-01-06

**Authors:** Shu-Ning Yan, Yi-Lei Wang, Annie Yu

**Affiliations:** 1 School of Fashion and Textiles, The Hong Kong Polytechnic University, Hong Kong, China; shu-ning.yan@connect.polyu.hk; 2 Faculty of Fiber Science and Engineering, Kyoto Institute of Technology, Matsugasaki, Sakyo-ku, Kyoto 606-8585, Japan; wyl9766@163.com

**Keywords:** cushioning, spacer fabric, square wave, inlaid, silicone tube, mechanical properties

## Abstract

Spacer fabrics are a breathable material option for wearable cushioning, but the cushioning performance is still not comparable to that of traditional elastomeric cushioning materials. The polymer-based connective structure of spacer fabrics largely affects fabric properties, compression, and mechanical performance, and this is a research gap that calls for the development of spacer fabrics with enhanced cushioning functions. This study develops a new square-wave inlay pattern and investigates the effects of the inlay structure and spatial frequency of the spacer course, as well as the effects of the silicone inlay on compression, impact force absorption, and vibration isolation of the spacer fabric. Twelve samples are designed and evaluated. The results show that the square-wave inlaid spacer fabric has higher energy absorption during compression. The square-wave pattern with a shorter transition distance between the front and back tuck stitches could increase the inclination angle close to a right angle, and extra tuck stitches on the surface float could secure the square-wave structure to enhance the impact force absorption ability. The increment in the spatial frequency of spacer courses provides a less stiff fabric with lower impact force absorption but higher vibration isolation ability. This study shows the innovative development of spacer fabric for enhancing cushioning properties.

## 1. Introduction

Cushioning materials are adopted in wearable cushioning products such as helmets, shoulder pads, elbow and knee braces, work gloves, bra cups, insoles, etc., to isolate vibration and absorb impact forces so as to protect the body from injuries. Elastomeric materials such as foam, PORON, ethylene-vinyl acetate (EVA), and neoprene are commonly used for wearable cushioning products and could be produced in various densities and stiffnesses and molded and cut into different shapes to fit end uses [1,2,3,4]. However, when used as a wearable product, the elastomeric foams restrict airflow and block convective heat exchange, thus causing poor air permeability and moisture transfer ability, which leads to thermal buildup, the trapping of sweat, and, hence, wear discomfort [5].

Knitted spacer fabric is a breathable alternative that can provide cushioning for wearable protective products, such as helmets, knee pads, insoles, sports bras, etc. [6,7,8,9,10]. Three-dimensional (3D) spacer fabrics have good air permeability due to their composition of knitted loops [11,12]. The polymer-based monofilament spacer yarns, which connect two knitted layers to form a 3D structure, provide a spring-like effect and absorb energy and cushion. Conventional weft-knitted spacer fabrics have surface layers made of a single jersey structure, and the connective layers are formed by several courses of polyester or polyamide monofilaments that are alternated as front and back tuck stitches with different needles to compose a net-like structure. The connective yarns and patterns can have immense influence on the fabric’s thickness, stiffness, compression, and energy absorption behaviors [13,14,15,16,17,18]. A thicker connective monofilament yarn can increase the stiffness of the fabric, while the connective pattern formed with a larger inclination angle to the surface can increase the fabric thickness and energy absorption [19,20]. However, the cushioning properties, such as impact force absorption of the spacer fabric, are still not comparable to those of elastomeric foam. Stacking several layers of spacer fabric could not only significantly improve cushioning [21] but also increase the bulk and reduce the feasibility of wearable protection. Shear-thickening fabric composites have been developed to enhance energy absorption abilities without a large increase in thickness [22,23,24,25]. However, the complicated process and inconsistent quality render these composites infeasible for commercial use. Previous studies suggest inlaying silicone tube materials into the connective structure [26]. The effect of different types of inlay materials, such as silicone tube materials, has also been studied and shows significant improvement in compression stiffness and energy absorption [27]. However, the inlaid patterns are either all miss stitches or alternating front and back tuck stitches in the connective layer, which means the characteristic and influence of other inlaid patterns have not been explored. There is, therefore, a research gap that needs to be further investigated, in which the cushioning properties of spacer fabric are enhanced through a new design of the inlaid pattern of silicone tubes.

In conventional weft-knitted spacer fabrics, each pair of front and back surface courses is followed by connective monofilament courses. Increasing the number of courses and the density of these monofilaments, which act as a spring, can improve load sharing and increase overall stiffness [28,29]. However, overly high monofilament density can overconstrain the structure, elevate internal stress, promote frictional locking, and reduce energy absorption and vibration damping. Therefore, the strategic placement of spacer monofilaments and inlay materials can be carried out to adjust the effective spring rate and loss factor, thereby optimizing the vibration isolation and energy absorption of the fabric. Since there are no studies that examine this issue, it is important to systematically investigate the density, spatial frequency, and placement pattern of both monofilament spacer yarns and inlay materials in weft-knitted spacer fabrics.

This study examines the inlay pattern of silicone tubes in spacer fabric and investigates the effects of the spatial frequency of both the monofilament and inlaid courses on compression, impact force absorption, and vibration isolation properties. The findings can provide design guidelines for spacer course proportion and inlay placement to meet the target protective function to enhance the performance of knitted cushioning.

## 2. Materials and Methodology

### 2.1. Square-Wave Silicone Inlaid Design

**Figure 1:**
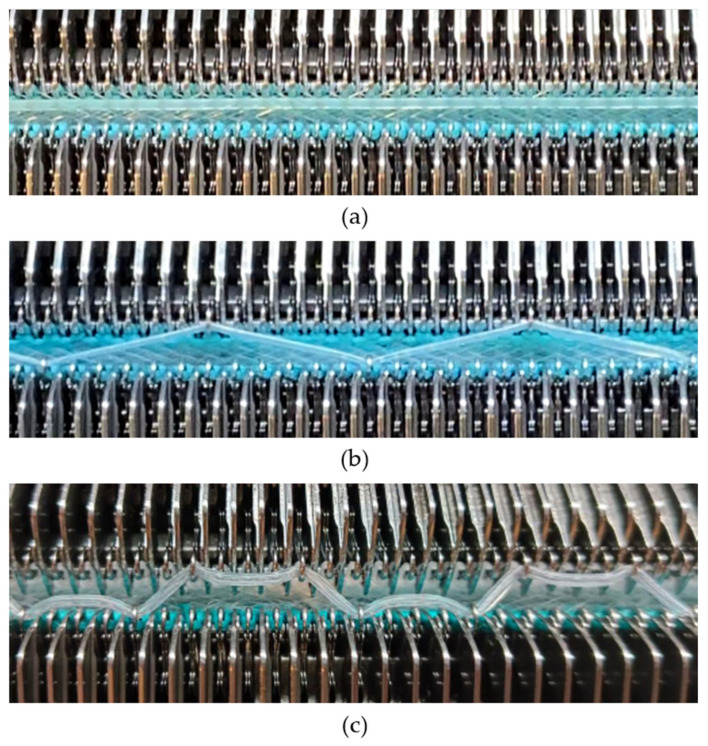
Spacer fabrics knitted by using a V-bed weft knitting machine with silicone inlaid in the form of (**a**) float yarn, (**b**) a zig-zag pattern, and (**c**) a square-wave pattern.

A square-wave pattern for silicone inlay was developed in this study. The silicone inlay used previously was either as a float yarn formed by all miss stitches in the connective layer (Figure 1a) or as a zig-zag pattern formed by alternating front and back tuck stitches, similarly to that of spacer yarn courses (Figure 1b). In order to explore ways to enhance the cushioning properties of spacer fabric, a new silicone inlay method was developed. The square-wave pattern used was inspired by architectural elements, that is, a bridge deck, to increase the stiffness without adding excessive mass and to provide local buckling restraint. Silicone tubes are used to form the square-wave pattern in the connective layer to reinforce the fabric structure (Figure 1c). The steps used help distribute forces, reduce stress concentration on the tuck stitches, and provide lateral stability. The multiple angles and surfaces can dissipate and absorb energy from impact.

### 2.2. Samples

**Figure 2:**
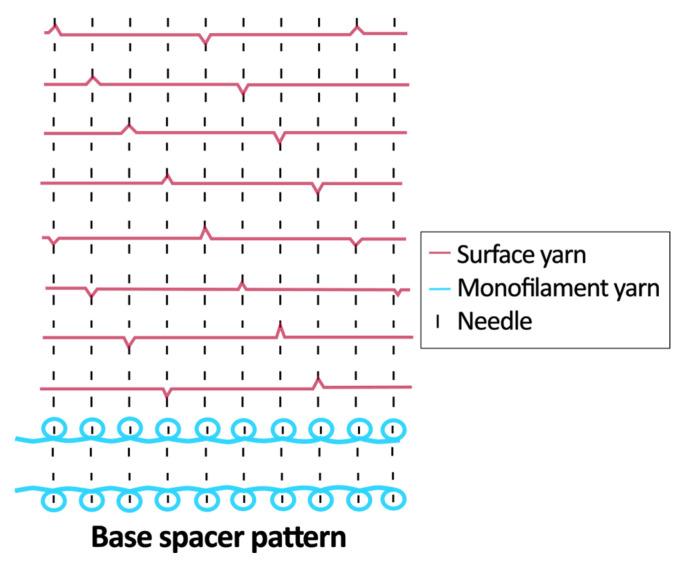
Base spacer pattern used by all samples.

Twelve spacer fabric samples were developed using a V-bed weft knitting machine (SWG091N210G, Shima Seiki, Wakayama, Japan). All samples were constructed using the same polymeric yarns and materials and based on the same base spacer pattern. Polyester drawn textured yarn (450D) and spandex yarns (140D) were used to form the front and back surface courses. The spacer courses were formed using polyester monofilament with a diameter of 0.12 mm. Silicone hollow tubes with a 1 mm tube diameter and 0.5 mm hollow diameter were used as the inlay material. A base spacer structure with three needles in between the front and back tuck stitches, 8 courses of spacer yarns, 1 front course, and 1 back course was adopted as the spacer base pattern. The spacer base pattern is shown in Figure 2, in which blue color indicates the surface yarns and red color indicates the monofilament spacer yarns. The twelve samples involved four main groups, SW, ST, FL, and B, representing square-wave inlay, square-wave inlay with an additional tuck stitch on horizontal float, float inlay, and a basic spacer without inlay, respectively.

Six samples (SW1, SW2, SW3, ST1, ST2, and ST3) were constructed with the square-wave-shaped silicone inlay. The silicone inlay was bound by a set of surface yarn and used after every three repeats of the spacer base pattern. To create a balanced structure, the square-wave pattern of the inlay appeared to be reciprocal in the front and back tuck stitches in alternative inlaid courses. The 6 square-wave samples were developed with variations in two parameters, which are the distance between the transition of the front and back tucks and the presence of an additional tuck stitch between two consecutive front tucks or two consecutive back tucks. The transition distance refers to the spacing between the front and back tucks within the inlay, measured by the number of needles separating them. Specifically, SW1 and ST1 featured one-needle spacing, SW2 and ST2 had two-needle spacing, and SW3 and ST3 incorporated three-needle spacing between the tucks. The transition distance for the front and back tucks could affect the inclination angles of the square-wave pattern, while the additional tuck stitch could influence the geometry of the square-wave inlay after the spacer fabric is removed from the knitting machine.

Another 3 samples with the silicone inlaid into the connective layer in a float pattern using all miss stitches were produced. Sample FL1 with the float of the silicone tubes bounded by three surface courses and applied after every three repeats of the base pattern was used as a comparison of the effect of the square-wave inlaid pattern. In order to investigate the effect of the spatial frequency and placement pattern of inlaid and monofilament yarns, two samples (FL2 and FL3) with different spacer-yarn-to-surface-course proportions and inlaid densities were developed. FL2 has a float of silicone inlay bounded by 3 surface courses after every repeat of the base pattern, while FL3 has a float of silicone inlay bounded by 5 surface courses after every repeat of the base pattern. Three corresponding samples (B1, B2, and B3) without inlay were also developed for comparison purposes and to evaluate the effect of the spacer yarn on the surface yarn proportion. The constructions in the form of a yarn path diagram of the 12 samples are shown in Table 1.

### 2.3. Evaluation

In order to understand the cushioning properties of the samples, fabric thickness, density, air resistance, compression behavior, force absorption, and vibration isolation properties were evaluated. Air resistance was tested using an air permeability tester (KES-F8, Kato Tech Co., Ltd., Kyoto, Japan) with an air vent area of 6.281 cm^2^ and an air volume of 2 cm/s. Five points of each sample were measured. The compression behavior was measured using a compression tester (EZ-S, Shimadzu, Kyoto, Japan) with a circular compression area of 109.36 cm^2^ (11.8 cm in diameter). The three test specimens for each sample were prepared in a circular shape with a diameter of 9 cm and compressed at a constant rate of 12 mm/min to the maximum compression stress of 460 N. A compression pressure of around 72 kPa was given to the sample, which reflects the realistic localized interface pressure in practical use, such as seating or footwear, and aligns with the force ranges of the compression test ASTM D3574-17: Standard Test Methods for Flexible Cellular Materials—Slab, Bonded, and Molded Urethane Foams [30]. The compression energy was calculated as the area under the compression stress–strain curve. The impact force absorption was evaluated using a ball drop test in accordance with ASTM D2632-15(2024): Standard Test Method for Rubber Property—Resilience by Vertical Rebound [31]. The experimental setup consisted of a dynamic load cell (Dytran series 1051, Dytran Instruments, Inc., Chatsworth, CA, USA) mounted at the bottom and positioned beneath two plies of the test specimens. A ball bearing was then dropped from a tube at a height of 400 mm onto the samples. Ten randomly selected locations of each sample were tested. The maximum impact force and reaction time of the materials were subsequently recorded. The force reduction (FR) percentage was calculated using the following:

FR (%) = (1 – F_x_/F_o_) × 100% (1)

where F_x_ is the peak force (N) of the tested samples, and F_o_ is the peak force of the ground surface (N). The vibration transmissibility was evaluated in accordance with ISO 13753:2008: Mechanical vibration and shock. Hand-arm vibration. Method for measuring the vibration transmissibility of resilient materials when loaded by the hand-arm system [32]. The test specimens were also prepared in a circular shape with a diameter of 9 cm. Following Wang et al. [33], a wide-band random signal vibration with a frequency range of 20–1000 Hz and an excitation magnitude of 1 m/s^2^ was supplied to the test platform for placing the specimen. A cylindrical load mass with a weight of 2.5 kg and a diameter of 9 cm was placed on top of the samples. An accelerometer (a_1_) placed at the center of the test platform and another accelerometer (a_2_) positioned at the top and center of the load mass were connected to a fast Fourier transform (FFT) analyzer (CF-9200, Ono Sokki, Japan) to examine the vibration transmissibility (T) in terms of the magnitude of the frequency response using a frequency response function (FRF):

(2) $$T=20{log}_{10}\left|\left.\frac{A_{2}}{A_{1}}\right|\right.$$

where A_1_ and A_2_ are the Fourier transforms of a_1_ and a_2_, respectively. FRF characterizes the steady-state response to the input signals, and the magnitude of the FRF result indicates the amplitude of the output signal compared to the input signal.

### 2.4. Analysis

Statistical analyses were carried out using SPSS 28 software (SPSS Statistics for Windows, Version 28.0, IBM Corp., Armonk, New York, USA). One-way analysis of variance (ANOVA) was used to analyze the impact of the fabric types on different fabric properties, including fabric thickness, stitch densities, air permeability, compression energy, and FR. A Bonferroni pairwise test was used to evaluate the effect of two samples. Pearson’s correlation coefficient was also used to analyze the relationship between fabric density and air permeability. The alpha level was set at 0.05 for statistical significance.

## 3. Results and Discussion

### 3.1. Effect on Physical Properties

**Figure 3:**
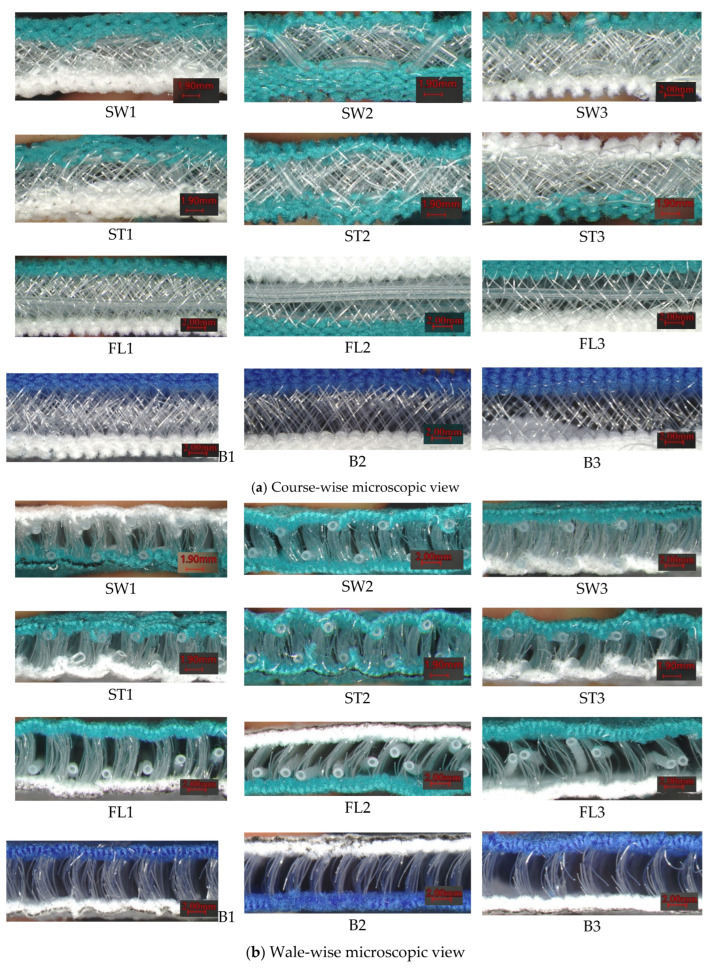
Microscopic views of samples: (**a**) course-wise and (**b**) wale-wise cross-sections.

Figure 3 presents the microscopic images of the twelve fabric samples in both the course-wise and wale-wise directions. It can be observed in the course-wise cross-section that the inclination angles and geometry of the silicone inlay vary due to the inlay pattern. In the wale-wise cross-section, it can be observed that the reciprocal square-wave pattern of the silicone inlay contracts the fabric through the thickness direction and, hence, creates a wavy fabric surface.

**Figure 4:**
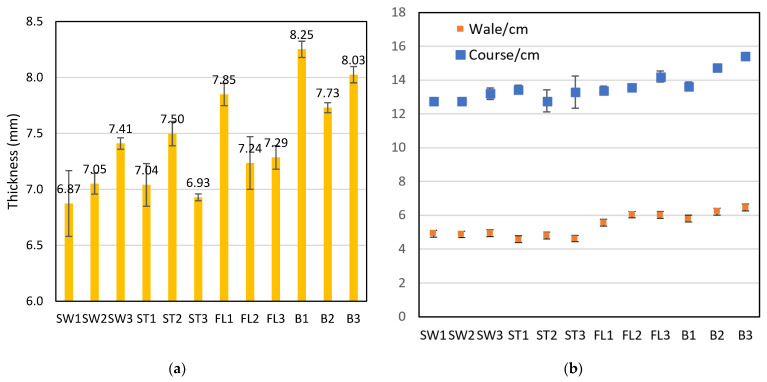
(**a**) Thickness and (**b**) wale and course density of the twelve fabric samples.

The thickness of the samples ranges from 6.87 to 8.25 mm (Figure 4). B1 had a significantly greater thickness than all other samples, except for B3. B1 had a higher spacer course density than B2 and B3, which can support the 3D structure and offer more thickness. The samples without the silicone inlay (B1, B2, and B3) had a significantly higher thickness than the corresponding samples with a silicone inlay (FL1, FL2, and FL3). The presence of a silicone inlay in the spacer fabric structure could affect the relaxation of the fabric after removal from the knitting machine and, hence, the fabric’s thickness. SW1, SW2, ST1, and ST3 all have thicknesses below 7.05 mm, so they are relatively less thick. The silicone tube has certain elasticity, being straightened and under tension during the knitting process. When the fabric was taken out of the knitting machine and allowed to relax, the elastic silicone tube would contract. The vertical transition of the square-wave inlay could contract the fabric structure through the thickness direction and hence reduce the thickness. Among the square-wave pattern inlaid samples, SW3 and ST2 are significantly thicker. The longer length of the elastic silicone tube in the transition of the front and back tucks leads to the contraction of the fabric along the course direction and increases the thickness. The extra tuck stitches with the surface prevent ST3 from undergoing further contraction, resulting in a lower thickness than that of ST2.

**Figure 5:**
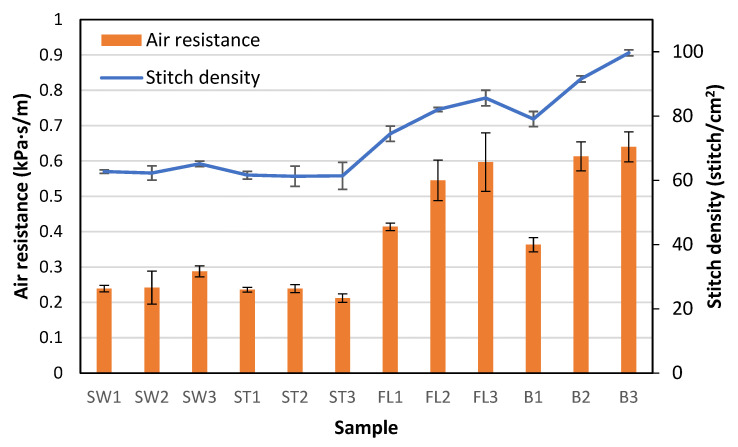
Air permeability and stitch density of the fabric samples.

Air permeability could affect the wear comfort of a wearable protective device. It was found that the samples with the square-wave pattern had relatively lower air resistance, which means higher air permeability for facilitating breathability. A 2.15 mm laminated polyurethane foam was measured to have an air permeability of 15.5 mL/s/cm^2^ at 100 Pa [34] converted to an air resistance of 0.65 kPa·s/m, which is higher than all fabric samples in this study with triple thickness. This confirms the breathability of the spacer fabric over polyurethane foam materials. Pearson’s correlation coefficient shows that there are significant correlations between air resistance and wale density (r = 0.938), course density (0.799), and stitch density (r = 0.941). The relationship is shown in Figure 5. It can also be observed that B1 has a significantly lower stitch density and air resistance than B2 and B3. FL1 also has a significantly lower stitch density and air resistance than FL2 and FL3. B1 and FL1 have a relatively higher proportion of the base spacer pattern in the structure. The spacer structure created with tuck stitches using the monofilament yarn affects the spacer fabric’s geometry, stitch density, and, finally, air resistance. The samples with the silicone inlay in a square-wave pattern have significantly lower air resistance than the samples with the inlay in a float pattern or no inlay. To form the square-wave pattern, the silicone tube needs to form tuck stitches on both the front and back surfaces. The tuck stitches of the silicone reduce both the wale and course densities, which facilitates the passage of air.

### 3.2. Effect on Compression Performance

**Figure 6:**
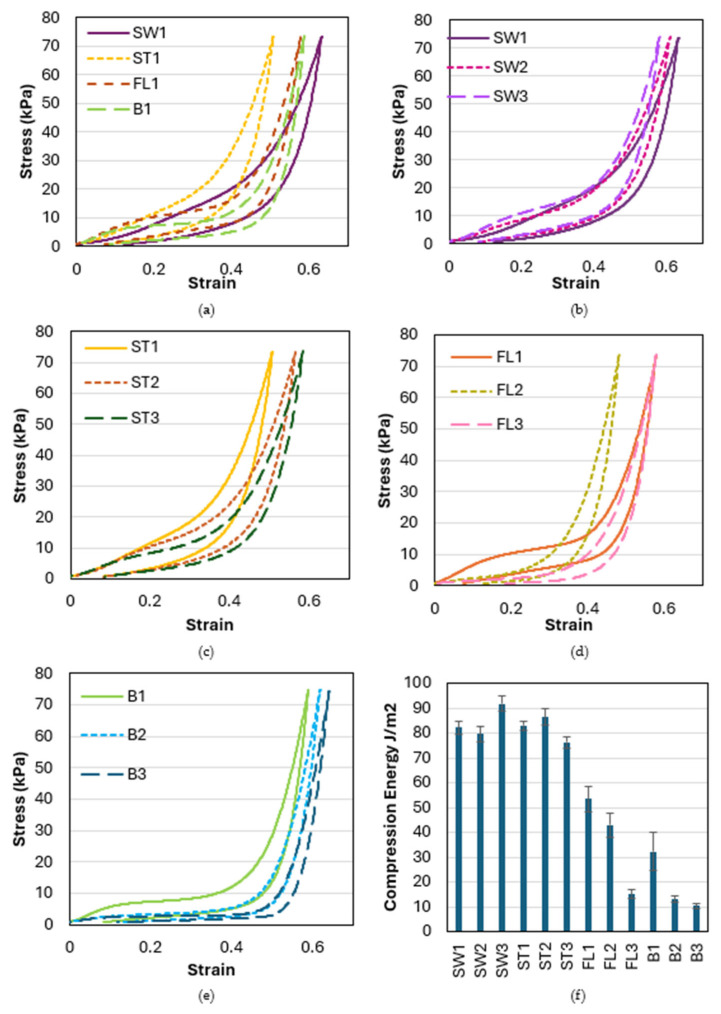
Compression stress–strain curve of samples with (**a**) different inlaid patterns, (**b**) square-wave inlaid pattern with different transition distances, (**c**) square-wave inlaid pattern with an additional tuck stitch on the horizontal float with different transition distances, (**d**) float inlaid with different inlaid and spacer densities, (**e**) no inlay with different spacer densities, and (**f**) compression energy of all samples.

The compression stress–strain curve and the compression energy of each sample are presented in Figure 6. The effect of the inlay structure can be observed by comparing SW1, ST1, FL1, and B1 (Figure 6a). The fabric stiffness and energy absorption of B1, which does not have the silicone inlay, are the lowest amongst the four samples. This confirms the findings of a previous study [26] in that the silicone inlay can help increase fabric stiffness and energy absorption. SW1 and ST1 show significantly higher compression energy absorption than FL1. FL1 shows a plateau in the stress–strain curve, which is commonly found in spacer fabrics due to buckling or shearing of the monofilament spacer yarns [20]. On the other hand, SW1 and ST1 maintain the initial fabric stiffness, which can be explained by the slope of the stress–strain curve, showing a higher compression strain without an obvious plateau. The square-wave inlay pattern of SW1 and ST1 reinforces the fabric structure and provides better support against compression. With the extra tuck stitch added between the two front or back tucks to maintain the square-wave geometry, the compression stiffness of ST1 becomes higher than that of SW1.

**Figure 7:**
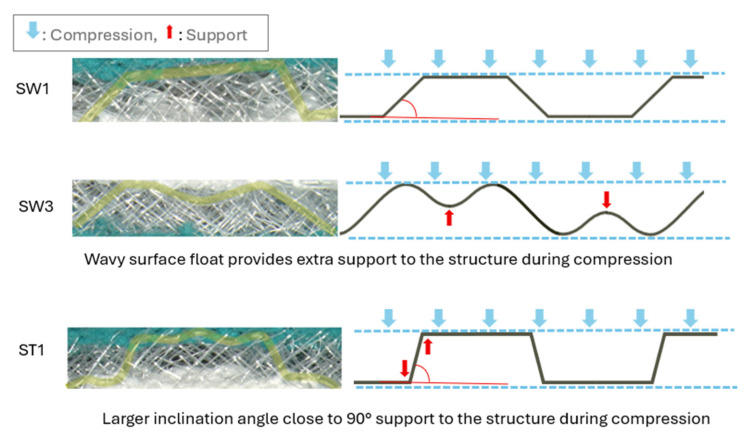
Comparison of the geometry of inlaid silicone with different transition distances of front and back tucks (SW1 and SW3) and with a different tuck pattern on the surface (SW1 and ST1).

The impact of the distance between the transition of the front and back tucks on the square-wave inlaid fabrics, SW1, SW2, and SW3, is very small until the compression strain reaches 0.4 (Figure 6b). With a compression strain above 0.4, the fabric stiffness increases when the transition distance increases. A float of silicone tube is formed along the fabric surface with the square-wave pattern. With an increase in the transition distance between the front and back tucks, which facilitates the contraction of fabric in the course-wise direction, the long float of the silicone curves and becomes a wavy structure, which is found to increase fabric stiffness during the contraction stage (Figure 7).

With the additional surface tuck of the square-wave silicone inlay, ST1, ST2, and ST3 show similar stress–strain behavior when the strain increases from 0 to 0.1 (Figure 6c). When the compression strain becomes higher, the fabric stiffness decreases as the transition distance increases. As the silicone tube floats along the fabric surface are fixed by the extra tuck stitch, the fabric’s contraction along the course-wise direction is mainly concentrated in the area where the silicone tube transitions between the two fabric surfaces. With the shorter distance of the transition of the front and back tucks, the inclination angle of the silicone tube inlay across the connective layer is reduced. The square-wave structure is stiffer and more resistant to compression when the inclination is closer to a right angle, thus providing optimal support through the thickness direction. When the transition distance is increased, the impact of the extra tuck stitch on the fabric’s stiffness diminishes.

By looking at samples with different inlaid densities (FL1, FL2, and FL3, as shown in Figure 6d), and different spatial frequencies of the spacer yarn (B1, B2, and B3, as shown in Figure 6e), a significant reduction in fabric stiffness and amount of energy absorption can be observed in reducing the proportion of monofilament spacer connection to the surface course. The spacer yarn not only connects the two surfaces but also forms the core for supporting the structure. Therefore, the fabric becomes easier to compress to a larger strain when the proportion of spacer yarn is reduced. The spacer yarn acts as the basis for supporting the structure, while the silicone inlay acts as the reinforcement.

### 3.3. Effect on Force Absorption

**Figure 8:**
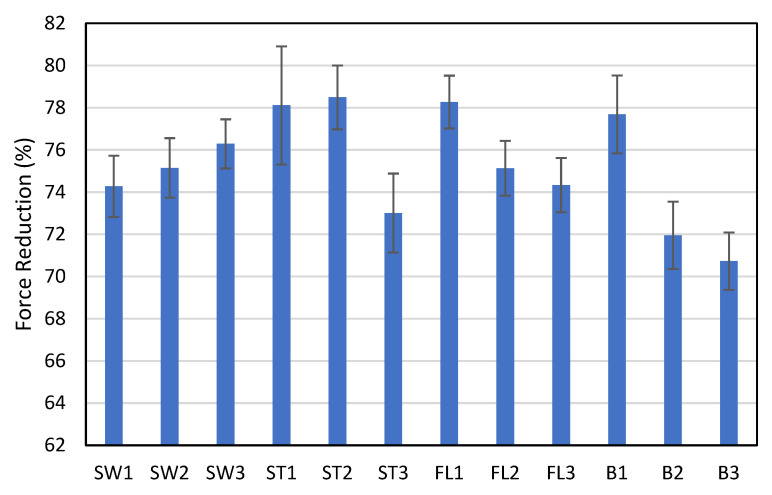
Force reduction percentage of the samples in the ball-drop test.

The FR % of the fabric samples is presented in Figure 8. ST2, ST1, and FL1 show relatively high FR of over 78%. In terms of the impact force absorption, the square-wave inlay pattern does not show many advantages over the float yarn inlay. However, the fabric thicknesses of the square-wave inlay samples are significantly lower than FL1, which indicates that ST1 and ST2 can have FR and FL1 with reduced thickness. ST1 and ST2 have higher FR than SW1, SW2, and SW3. The extra front and back tuck stitches of the square-wave inlay have the potential to better reduce impact force. It is interesting to note that ST3 offers the lowest FR amongst the inlay samples. The increase in the transition distance between the front and back tuck stitches of the square-wave pattern with extra tuck stitches diminishes impact force absorption ability. B3 offers significantly lower FR than all other samples (*p* < 0.05). FL1 and B1 also show a significantly higher FR than FL2, FL3, B2, and B3. The reduction in the proportion of monofilament spacer connections not only provides a less stiff fabric but also significantly lowers the impact force absorption ability.

### 3.4. Effect on Vibration Isolation

**Figure 9:**
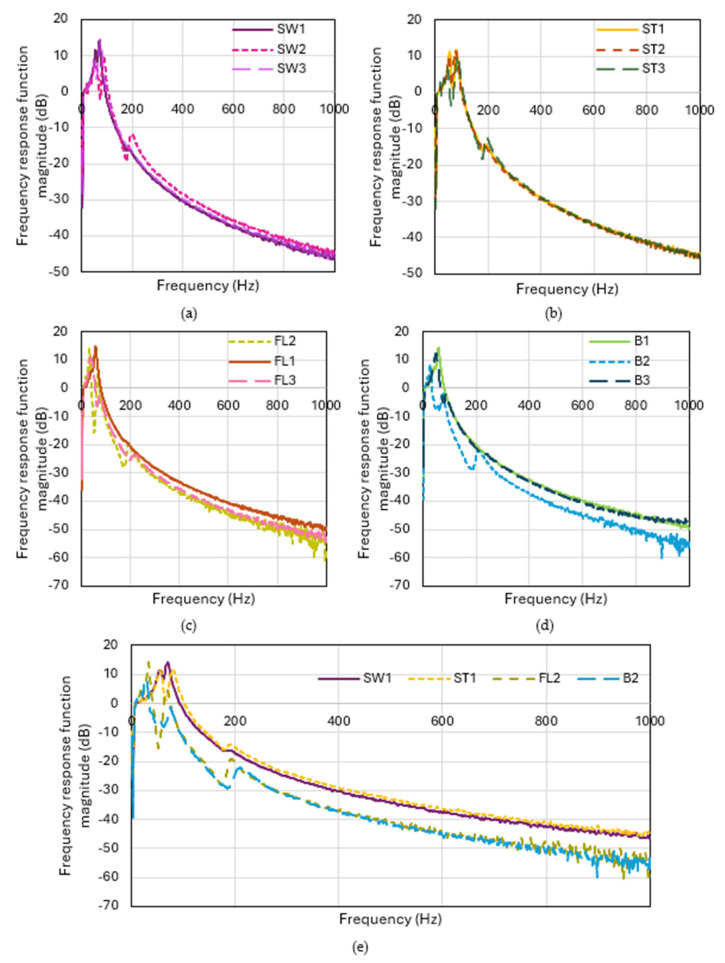
Vibration transmissibility at 0–1000 Hz of the samples with (**a**) square-wave inlaid pattern with different transition distances, (**b**) square-wave inlaid pattern with an additional tuck stitch on the horizontal float with different transition distances, (**c**) float inlay with different inlay and spacer densities, (**d**) no inlay with different spacer densities, and (**e**) the optimal sample in each sample group.

The vibration transmissibility curves of the fabric samples at 0–1000 Hz are presented in Figure 9. An FRF below 0 indicates the frequencies at which the fabric acts as a filter to reduce vibration. The vibration transmissibility of the square-wave inlaid samples is quite similar. There is more than one peak on the FRF curve of the square-wave inlaid samples, thus indicating the presence of multiple resonant frequencies due to the dynamic mechanical behavior inside the structure. The square-wave inlay does not enhance the vibration isolation of the spacer fabric but increases the complexity of the vibration system, triggering multiple natural frequencies. The square-wave silicone inlay not only creates a periodically varying fabric stiffness, but the tuck stitches and inlay segments also couple with the spacer fabric structure, producing extra resonances. On the other hand, FL2 and B2 show better vibration isolation with a lower natural frequency and reduce the vibration for a wider range of frequencies. FL2 and B2 have better vibration isolation than FL1 and B1, which have the spacer courses placed more compactly together, leading to frictional locking. The higher spatial frequency of B2 forms a less stiff fabric, which provides better damping in the test. B3 shows a higher natural frequency and a smaller, wider range and degree of vibration isolation than B2. The lower proportion of the spacer course to surface course in the spacer fabric reduces the appearance of the spacer course along the fabric length, which is the damper of the vibration transmission system, thus leading to poor vibration isolation.

## 4. Conclusions

This study has designed spacer fabrics with a square-wave silicone inlaid pattern and different spatial frequencies of the placement pattern of spacer courses. Twelve fabric samples are fabricated with a V-bed weft knitting machine. The air resistance, compression behavior, force absorption, and vibration isolation properties of the samples are evaluated and compared. The key findings are as follows:

- The square-wave inlay not only forms a more compact fabric with lower thickness but also with lower stitch density and higher air permeability.
- The square-wave inlaid spacer fabric has higher energy absorption and fabric stiffness without a significant plateau stage shown on the compression stress–strain curve.
- A shorter transition distance between the front and back tuck stitches of the square-wave pattern with extra tuck stitches on the surface float could enhance impact force absorption.
- The increment in spatial frequency of the monofilament spacer connections could provide a less stiff fabric with significantly lower impact force absorption but higher vibration isolation ability.

The development of the spacer fabrics presented in this study not only shows a new development in knitted spacer fabrics but also provides a new option for cushioning, especially for wearable products that prioritize breathability and wear comfort. This study did not assess the durability or washability of the spacer fabrics, which are essential considerations for wearable cushioning applications. Future work will evaluate these properties using standardized laundering and durability protocols to determine long-term performance.

## Figures and Tables

**Table 1:** Yarn path diagrams of samples.

Sample Groups	Sample Yarn Path Diagram		
SW—square-wave inlay	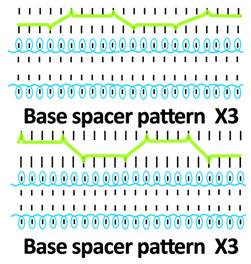 **SW1**	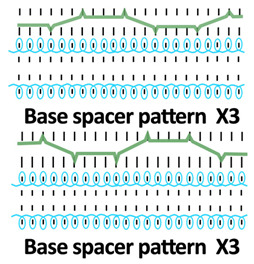 **SW2**	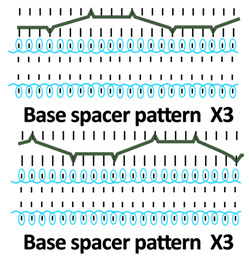 **SW3**
ST—square-wave inlay with addition-al tuck stitch on horizon-tal float	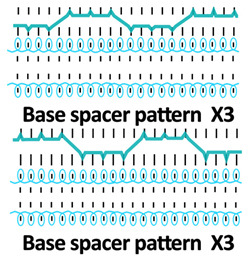 **ST1**	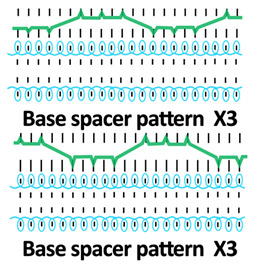 **ST2**	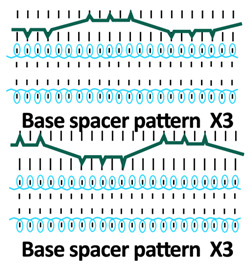 **ST3**
FL—float inlay	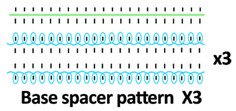 **FL1**	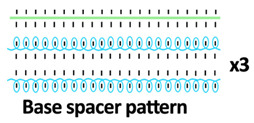 **FL2**	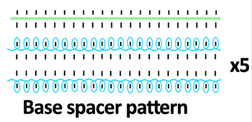 **FL3**
B—basic spacer without inlay	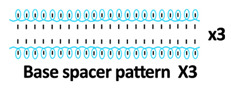 **B1**	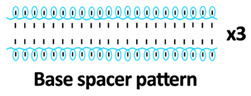 **B2**	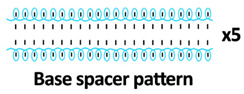 **B3**

Note: Blue color denotes the surface yarn with 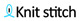, while green color represents the inlaid yarn with 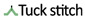 and 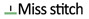.

## Data Availability

The raw data supporting the conclusions of this article will be made available by the authors upon request.
